# Segmentation by recreation experiences of demand in coastal and marine destinations: A study in Galapagos, Ecuador

**DOI:** 10.1371/journal.pone.0316614

**Published:** 2025-01-10

**Authors:** Mauricio Carvache-Franco, Lidija Bagarić, Orly Carvache-Franco, Wilmer Carvache-Franco

**Affiliations:** 1 Universidad Bolivariana del Ecuador, Durán, Ecuador; 2 Faculty of Tourism and Hospitality Management, University of Rijeka, Opatija, Croatia; 3 Universidad Espíritu Santo, Samborondón, Ecuador; 4 Facultad de Ciencias Sociales y Humanísticas, Escuela Superior Politécnica del Litoral, ESPOL, Guayaquil, Ecuador; Centro de Investigaciones Biologicas del Noroeste SC, MEXICO

## Abstract

Tourism in coastal and marine areas offers a wide variety of recreational activities. The present study had the following objectives: (i), identify the dimensions of recreational experiences in coastal and marine destinations focused on island marine protected areas (ii) determine the demand segments for recreational experiences, and (iii) establish the relationship between the demand segments for recreational experiences and the satisfaction and loyalty. The study was conducted in the Galápagos Islands of Ecuador, and 407 valid questionnaires were collected on-site. The statistical techniques used were factor analysis and the non-hierarchical k-means clustering method. The results reveal four dimensions of the recreational experience in coastal and marine destinations focused on island marine protected areas: Ecological Observation, Experiential Learning, Experiential Reflection, and Sensory Experience. Additionally, three demand segments differentiated by recreational experiences were identified: The Estheticians, the Multi-Experience Recreationists, and the Hedonists. Among these groups, the Multi-Experience Recreationists segment was the most satisfied and exhibited higher levels of loyalty in insular protected marine areas. These findings will assist managers of protected marine areas in creating sustainable development plans and contribute to the academic literature on coastal and marine tourism.

## 1. Introduction

Coastal and marine tourism in marine protected areas has become increasingly popular in recent years, attracting millions of visitors to destinations worldwide. Recreational experiences and activities play a vital role in this tourism typology, providing opportunities for visitors to engage with the marine environment and participate in various activities. One of the main attractions of coastal and marine destinations is their various recreational experiences and activities. National parks play an important role in global leisure activities and biodiversity conservation. They provide unique recreational experiences while protecting ecosystems and offering visitors the opportunity to engage in outdoor activities to create a deeper connection with nature. The blend of recreation and conservation efforts ensures that visitors not only enjoy their experience, but also contribute to the preservation of these natural landscapes. Effective park management that focuses on striking a balance between visitor satisfaction and environmental sustainability is crucial to ensuring that these protected areas remain resilient and continue to provide benefits for future generations. The unique combination of recreation and education in national parks supports the conservation of biodiversity while promoting environmental awareness among visitors.

In this case, coastal destinations are defined as areas with tourist activities based on coastal land, such as swimming, surfing, sunbathing, and other coastal leisure, recreational, and sports activities along the shores of a sea, lake, or river. Proximity to the coast is also a requirement for the services and facilities that support coastal tourism. Meanwhile, maritime tourism includes activities based on the sea, such as cruises, sailing on yachts or boats, and nautical sports and their respective onshore services and infrastructures.

Marine protected areas, part of the same industry as coastal and marine tourism, have recently experienced significant growth and have become popular destinations for travelers seeking peace and tranquility in nature. Marine protected areas are coastal and oceanic areas with unique flora and fauna. They are subject to regulations that govern activities and uses by both the public and private sectors, such as fishing, to ensure the conservation and sustainability of the area [1]. When well-managed, they effectively protect habitats and natural species from various local stressors, such as destructive fishing and pollution [2]. Marine protected areas are the preferred tool for preventing the loss of marine biodiversity [3]. They are also a nature-based tool for repairing environmental damage and maintaining a wide range of services provided by marine ecosystems [4].

Marine protected areas have been established globally to conserve marine ecosystems and biodiversity. Although the primary goal is conservation, they also provide opportunities for various recreational activities. In this literature review, the authors will discuss the importance of recreation and leisure activities and how they can impact conservation efforts. Coastal and marine tourism is a fast-growing sector of the global tourism industry. Understanding the dimensions or factors contributing to the recreational experience is essential to managing and developing tourism activities in these areas.

Despite the growing popularity of national parks as key destinations for leisure and recreation, there remains a significant research gap in understanding visitor segmentation based on recreational experiences in these protected areas. While many studies have examined general tourism trends and conservation efforts, few have looked specifically at how different types of visitors experience and interact with national parks. This lack of research makes it difficult to develop tailored management strategies that can address the different needs, preferences and behaviours of visitors. Without a clear understanding of visitor segments, park managers may struggle to implement effective strategies that increase visitor satisfaction while promoting sustainable practises. Furthermore, this gap limits the advancement of theoretical frameworks in tourism and conservation research, where understanding the nuances of recreation experiences is critical to both academic discourse and practical application. By focussing on this under-researched area, this study not only addresses a key deficit in the literature, but also provides valuable insights that can improve park management and contribute to the sustainability of national parks worldwide.

In this context, in South America are the Galápagos Islands, which form an archipelago in Ecuador in the Pacific Ocean. These islands were declared a World Natural Heritage Site in 1978. The arrival of tourists encourages coastal leisure recreational activities such as walks on the beach, flora and fauna watching, and whale watching. On the other hand, sports-related recreational activities encompass surfing, diving, snorkeling, recreational fishing, and sea kayaking.

Within this order of ideas, there are few studies of recreational experiences and activities in coastal and marine tourism in island marine protected areas in the literature review. Studies in this area could contribute to the growth and sustainability of marine protected areas. MPA. The present study has the following objectives: (i) identify the dimensions of recreational experiences in coastal and marine destinations focused on island marine protected areas, (ii) determine the demand segments for recreational experiences in coastal and marine destinations focused on island marine protected areas, and (iii) establish the relationship between the demand segments for recreational experiences and satisfaction and loyalty in coastal and marine destinations focused on island marine protected areas. The findings will contribute to management guides for administrators of marine protected areas and will be a contribution to academic literature in the area of coastal and marine tourism.

## 2. Literature review

### 2.1. Recreational experiences in coastal and marine destination

Recreational experiences in coastal and marine tourism can be broadly categorized as active or passive experiences. Active experiences include activities such as swimming, snorkeling, diving, surfing, and sailing, while passive experiences include activities such as sunbathing, beach walking, and sightseeing boating [5–7]. Recreational experiences in coastal and marine tourism provide visitors with opportunities to connect with nature, learn about local culture, and participate in physical activities, which can have essential health benefits [8–10]. These experiences can also provide emotional and psychological benefits, such as relaxation, stress reduction, and personal growth [5].

Coastal and marine tourism recreational activities can provide economic benefits by generating income for local businesses and communities [11]. However, if not appropriately managed, recreational activities can also have negative impacts on the natural environment and local communities [12,13].

Several management strategies can be developed to minimize the negative impacts of coastal and marine tourism recreational activities. They must balance the demands of recreational activities with conservation goals and community interests. For example, zoning, visitor restrictions, and environmental regulations have been implemented in many coastal and marine areas to regulate the number and location of recreational activities. In addition, education and awareness programs have been developed to inform visitors about the importance of protecting the natural environment and respecting local culture [14].

Experiences and recreational activities are essential for the success of coastal and marine tourist destinations. Recreational activities such as swimming, snorkeling, diving, and fishing attract visitors, thus generating income and publicity [6,15]. Marine protected areas provide a range of benefits for both visitors and tourist destinations, including economic, social, and environmental benefits. For example, recreational activities can generate income for local businesses, create employment opportunities, and contribute to the overall economy of the destination [16]. Furthermore, recreational activities can provide opportunities for cultural exchange and learning, promoting the social and cultural sustainability of the destination [17]. Because the literature is precise regarding the dimensions of the recreational experience in island marine protected areas; that is, studies have been conducted in other types of coastal destinations, and it is necessary to have evidence to contribute to the literature, our study poses the first research question: RQ1: What are the dimensions of the recreational experience in coastal and marine destinations focused on island marine protected areas?

### 2.2. Demand factors for recreational experiences in coastal and marine destination

Several different models have been proposed to describe the factors of the recreational experience in coastal and marine tourism. A commonly cited model is the four-factor model, which includes social, environmental, psychological, and physiological dimensions [18]. The dimensions or factors of the recreational experience in coastal and marine tourism can be measured and assessed using various methods, including surveys, interviews, and observations. A commonly used instrument is the Experience Sampling Method, which collects data on participants’ experiences at multiple points during the recreational activity using self-report measures. Another method involves using rating, or Likert scales to measure participants’ perceptions of various aspects of the experience, such as enjoyment, challenge, and satisfaction [19]. In another study, Kline et al. [20], in North Carolina, USA, identified four factors of the recreational experience: nature, local culture, local food, and corporate chains. In Acapulco, Mexico, the authors Carvache-Franco [10] identified four dimensions of recreational experiences, namely experiential reflection, experiential learning, ecological observation, and sensory and experiential.

Similarly, previous studies have identified various dimensions or factors influencing the recreational experience in coastal, marine, island, or marine protected areas. These include Environmental factors: The natural environment plays a crucial role in shaping the coastal and marine tourism experience. Water quality, marine biodiversity, scenic beauty, and climatic conditions significantly impact recreational activities and tourists’ overall experience. Newsome and Dowling [21] emphasize the importance of water quality and natural beauty for a satisfying tourism experience. The natural environment is an essential factor that shapes the recreational experience of coastal and marine protected area tourism. Elements such as scenic beauty, biodiversity, and water quality have been found to significantly influence visitors’ recreational experience [22].

Social Factors such as the presence of friends or family can also influence the recreational experience in coastal and marine tourism [23]. The social environment, including the presence of family members, friends, and other social groups, has been found to influence visitors’ recreational experiences in coastal, marine, island, or protected marine areas. Similarly, the presence of other visitors, the behavior of other visitors, and the level of crowding have been found to influence visitor experiences significantly [24], as well as sharing tourist experiences through social networks [25].

In this regard, personal factors have also been found to influence the recreational experiences of visitors in coastal and marine tourism. Age, gender, motivation, and previous experience have been discovered to influence recreational experiences [10,26]. Lee et al. [27] discuss the role of individual preferences and motivation in shaping the tourist experience, emphasizing the importance of understanding tourists’ emotional experiences. Öhman [28] also suggests that previous experience and risk perceptions are crucial personal factors influencing the recreational experience in coastal and marine tourism. The level of physical condition and the desire for adventure have also been identified as critical personal factors influencing visitor experiences in coastal and marine tourism [17]. Additionally, Lee et al. [27] on Liuqiu Island in Taiwan identified the following dimensions of recreational experiences: experiential sensory, experiential learning, experiential reflection, and ecological observation.

Cultural factors, such as the sea’s and the coast’s importance for local culture and heritage, can influence visitors’ experiences and behavior in coastal and marine tourism. Huh et al. [29] emphasize the importance of understanding a destination’s cultural context and social factors to create a satisfying tourist experience. The cultural environment of coastal and marine tourism areas also significantly shapes visitors’ recreational experiences. Heritage sites, museums, and other cultural attractions have been shown to significantly contribute to visitors’ satisfaction with their experiences [29,30]. Similarly, the availability of local food, crafts, and other products can also enhance the visitor’s experience [31,32].

Infrastructure factors on the availability and quality of infrastructure and facilities, such as accommodation, transportation, and recreational facilities, can also affect the tourism experience in coastal and marine areas. Rheeders [33], emphasizes the importance of the availability and quality of accommodation, transportation, and recreational facilities for coastal and marine tourism. Postma and Schmuecker [34] discuss the importance of tourism infrastructure and facilities to minimize negative impacts on the environment and local communities.

Management factors such as providing information materials can also affect visitor experiences [17] or the use of innovations and new technologies [35]. The variety and quality of tourism activities offered in coastal and marine areas can significantly influence the recreational experience. Lee et al. [36] discuss the importance of offering responsible and sustainable tourism activities to improve the recreational experience and promote the social responsibility of the destination.

Understanding the recreational experience’s dimensions or factors is crucial for managing and developing tourism activities in coastal and marine tourism. Administrators and planners can use this knowledge to design and develop activities that address visitors’ needs and preferences, ultimately increasing visitor satisfaction and promoting repeat visits [17,37]. Recreation administrators and planners can use this knowledge to design and develop activities catering to visitor needs and preferences. Furthermore, understanding the dimensions or factors of the recreational experience in coastal and marine tourism can help enhance visitor satisfaction and encourage repeat visits to recreational destinations [38].

### 2.3. Market segmentation for recreational experiences in coastal and marine destination

Market segmentation is dividing a market into distinct groups of consumers with similar needs or characteristics. This process enables marketing specialists to create targeted marketing strategies that cater to the specific characteristics of each segment. Market segmentation is essential for marketing success and can be systematized in various ways. The most commonly used approaches are based on organizational constraints and the choice of segmentation variables [39]. Market segmentation has become essential for understanding and guiding consumers based on their preferences, needs, and expectations [40]. In the recreational sector, segmentation by experience allows companies to provide personalized offerings that appeal to specific consumer groups, ultimately enhancing customer satisfaction and loyalty. In the context of coastal and marine tourism, market segmentation by recreational experiences refers to dividing the market of potential visitors into groups based on the types of recreational activities that interest them. This approach can help tour operators develop specific marketing strategies that meet the needs and preferences of specific tourist groups. Recreational experience is a multifaceted construct that encompasses several dimensions or factors. Dimensions of the recreational experience identified in the literature include authenticity, novelty, challenge, social interaction, relaxation, escape, and education [17,27,41].

In recent years, there has been a growing interest in understanding the market segmentation of tourists who visit coastal marine protected areas for recreational purposes. Several studies have investigated the preferences and behaviors of tourists in these areas. Ballantyne et al. [17] identified four levels of visitor response to coastal and marine tourism experiences: sensory impressions, emotional affinity, reflective response, and behavioral response. In another study on recreational segmentation in coastal and marine destinations, Almeida et al. [42], on the island of Madeira in Portugal, identified four groups of rural tourists: the relaxation segment, which included individuals looking to relax and recharge, was the most dominant, indicating that this sector mimics the primary tourism market. The ruralist group valued relaxation in natural settings as an escape from daily routine. The “I want it all” group included tourists interested in various activities, and the family-oriented group was attracted to family socialization. Lee et al. [27] proposed a segmentation model based on the dimensions of the recreational experience. In the study on recreational experience segmentation on Taiwan’s Liuqiu Island, they used a scale that included items examining experiential engagement (seven items), reflective engagement (six items), aesthetics (four items), and education (four items). They identified four distinct groups: (1) multiple experience recreationists, who enjoyed all aspects of recreational experiences compared to the other three groups. (2) Aesthetes who preferred experiencing beautiful landscapes with the highest scores in experiential aesthetics. (3) Hedonists who had lower levels of recreational experience than the other three groups. (4) Knowledge seekers, who were more likely to appreciate aesthetics and learning experiences. Scholars Carvache-Franco et al. [10] in Mexico identified four segments of recreational experiences in coastal and marine destinations: multiple-experience recreationists, hedonists, knowledge seekers, and aesthetes.

Based on previous findings, few studies have been carried out on segmentation by recreation in coastal and marine destinations. No previous studies have been found in island destinations with protected areas. Therefore, it would be imperative to determine the demand segments for recreational experiences in this type of protected island destination, all aligned with our second research question: RQ2: What are the segments of the recreational experience in coastal and marine destinations focused on island marine protected areas?

### 2.4. Relationship between segmentation by recreational experiences with satisfaction and loyalty in coastal and marine destinations

Tourist satisfaction is a critical factor in the success of the tourism industry since it can influence repeat visits, positive word-of-mouth, and the image of the destination [43–45]. Understanding the determinants of tourist satisfaction and the role of expectations and experiences is essential to improving service quality and enhancing the tourism experience [46]. Expectancy confirmation theory is a widely used framework to examine satisfaction in the context of coastal and marine tourism. According to this, satisfaction results from comparing expectations and perceived performance [47]. Tourists form expectations based on previous experiences, information from external sources, and personal needs and desires [48]. When perceived performance meets or exceeds expectations, tourists will likely be satisfied [49,50].

Tourist experiences are dynamic and multifaceted, encompassing various elements such as attractions, activities, accommodation, transportation, and interactions with locals and other tourists [51]. These elements can be classified into functional and emotional aspects. Functional aspects refer to the tangible components of a tourist experience, such as the quality of accommodations or the cleanliness of a destination. Emotional aspects involve the feelings and emotions evoked during a trip, such as relaxation, excitement, or cultural immersion [52]. Both functional and emotional aspects play an essential role in shaping tourist satisfaction. Functional aspects are directly related to the performance of various services and facilities, while emotional aspects contribute to a destination’s overall atmosphere and unique experience. A comprehensive tourist experience that meets or exceeds expectations in both functional and emotional aspects is more likely to generate higher satisfaction levels [53]. The relationship between expectations, experiences, and satisfaction can vary in contexts of coastal and marine tourism. For example, the relative importance of functional and emotional aspects in determining satisfaction may differ depending on the type of tourist, the purpose of the trip, and the specific destination [49,52].

Several factors influence tourist satisfaction, including destination attributes, service quality, personal factors, emotions, and perceived value for money. Destination attributes, including natural and cultural attractions, accommodation options, transportation infrastructure, and local cuisine, can influence tourist satisfaction [43,54]. Tourists are more likely to be satisfied when the destination offers a wide variety of high-quality attractions and services that cater to their needs and preferences. Service quality is a critical determinant of tourist satisfaction [55,56].

High-quality services, such as efficient check-in procedures, clean accommodations, and friendly staff, can significantly contribute to positive tourist experiences and overall satisfaction. On the other hand, poor service quality can lead to dissatisfaction and negative word-of-mouth. Personal factors, such as individual preferences, motivations, and cultural backgrounds, can also influence tourist satisfaction [57]. Tourists with different motivations, such as relaxation, adventure, or cultural exploration, may have different expectations and evaluations of their experiences. Additionally, tourists from different cultural backgrounds may have varying preferences and expectations regarding service quality, communication styles, and social norms, which can impact their overall satisfaction with a destination [54,58]. Tourists’ emotional experiences during their trip, such as joy, excitement, and relaxation, can affect their overall satisfaction [52,59]. Perceived value for money is another important factor that affects tourist satisfaction [44]. Tourists are more likely to be satisfied when they believe that the benefits they receive from a trip, such as memorable experiences and personal growth, outweigh the costs incurred, including travel expenses and time investment.

Several models have been proposed to measure tourist satisfaction, each with strengths and limitations. Based on the expectancy-confirmation theory, the expectation-disconfirmation model posits that satisfaction is determined by the discrepancy between pre-travel expectations and perceived post-travel performance [47,60]. Positive disconfirmation occurs when perceived performance exceeds expectations, resulting in satisfaction, while negative disconfirmation occurs when perceived performance falls short of expectations, leading to dissatisfaction. This model is widely used in tourism research due to its simplicity and ability to capture the dynamic relationship between expectations and experiences. The Service Quality (SERVQUAL) model, developed by Parasuraman et al. [61], is another popular approach to measuring tourist satisfaction. This model assesses service quality across dimensions. By measuring the gap between tourists’ expectations and perceptions, the SERVQUAL model helps identify areas of improvement and enhance overall satisfaction. By mapping destination attributes onto this grid, tourism industry stakeholders can prioritize improvement areas based on the importance of attributes to tourists and their current performance [62–64].

Understanding tourist satisfaction is essential to the continued growth and success of the coastal and marine tourism. Researchers and practitioners can develop strategies by examining the interaction between expectations and experiences and considering factors influencing satisfaction.

Loyalty in the tourism industry has become increasingly important as organizations strive to retain customers and maintain a competitive advantage [65]. In tourism, loyalty is often operationalized in terms of tourists’ intentions to revisit a destination, their willingness to recommend it to others, and their overall satisfaction with the coastal and marine tourism experience [66]. Some researchers have also proposed segmenting tourists based on their levels of loyalty, distinguishing between first-time visitors, repeat visitors, and loyal visitors [67].

Tourist characteristics such as sociodemographic factors (age, gender, income, education), travel motivations, and previous experience have been found to influence loyalty [43]. For example, tourists with higher income levels and more travel experience are more likely to be loyal to a destination [68]. Destination attributes affecting loyalty include destination image, perceived quality, value, and satisfaction [69]. A positive destination image and high satisfaction levels are consistently strong predictors of loyalty [70]. External influences such as the role of marketing efforts, social media, and word-of-mouth communication can also impact loyalty [71]. For instance, tourists who rely heavily on word-of-mouth recommendations are more likely to be loyal to a destination [72]. Loyal tourists are valuable to organizations and tourist destinations since they exhibit positive behaviors such as repeated visits, positive word-of-mouth communication, and lower price sensitivity [73]. Additionally, loyal tourists contribute to a destination’s economic stability and long-term growth by providing a stable source of income and reducing the marketing costs associated with attracting new visitors [74].

A variety of destination attributes and tourist motivations influence tourist loyalty. Understanding these factors can help tourism stakeholders develop strategies to enhance loyalty and encourage repeated visits in coastal and marine areas. Destination attributes such as natural beauty, cultural attractions, infrastructure, and service quality can significantly impact tourist loyalty. Research has shown that destinations with a strong and unique image are more likely to attract loyal tourists [70]. Furthermore, high satisfaction levels with various destination attributes can lead to greater loyalty [69]. Tourism stakeholders should invest in improving and promoting the unique attributes of their destination to increase tourist loyalty. Tourist motivations, such as relaxation, adventure, social interaction, and personal development, can also affect loyalty. Tourists who perceive that a destination meets their motivational needs are likelier to become loyal visitors [68]. Therefore, tourism stakeholders should focus on understanding the motivations of their target market and develop products and services that meet these needs.

Satisfaction and emotional attachment are critical factors that mediate the relationship between destination attributes, tourism motivations, and loyalty. High satisfaction with destination attributes and a solid emotional attachment to a destination can lead to increased loyalty [66]. In the study by Carvache-Franco et al. [10], the academics found that the recreationists with multiple experiences segment were the most satisfied and loyal to the destination. Tourism stakeholders should create memorable experiences that foster emotional connections and improve overall satisfaction to promote tourist loyalty. Since no previous studies have been found that measure the relationship between demand segments and tourist satisfaction and loyalty in island-protected destinations, we pose our third research question: RQ3: What is the relationship between recreational experience segments and satisfaction and loyalty in coastal and marine destinations focused on island marine protected areas?

## 3. Study area

Galápagos is an archipelago and province of Ecuador, covering an area of 8,010 square kilometers in the Pacific Ocean. It was declared a UNESCO World Heritage Site in 1978. In March 2023, it recorded the arrival of 32,000 tourists, which was 24% more than in 2019 [75]. The archipelago comprises 13 islands more extensive than 10 square kilometers and over 200 islets. San Cristóbal, Fernandina, Isabela, Santiago, Floreana, Santa Cruz, and Marchena are the most relevant islands. Galápagos is characterized by its vibrant diversity of flora and fauna, being the home to endemic species and having a variety of unique geological formations. Recreational coastal tourism activities include whale watching and observing other marine species, as the Galápagos Archipelago Marine Reserve has documented approximately 23 cetacean species. Due to its potential for observing flora and fauna, 71 terrestrial visitor sites have been recognized, including areas with colonies of seabirds such as blue-footed boobies and frigatebirds. There are various travel options by boat in Galápagos, typically lasting from 4 to 15 days on average. These trips aim to explore various parts of the archipelago and make stops at specific points to allow tourists to explore. Due to its coral reefs, exploration and beach walks are common on Isabela Island.

Among the recreational activities related to sports, surfing stands out on San Cristóbal Island (the most recommended points are Tongo Reef, Cañon Carola, and La Lobería). This activity can be carried out throughout the year. However, it is recognized that the best season is from December to May. Diving is characterized by the sighting and exploration of reefs and interaction with species of sharks, turtles, and whales. Kayaking is intended to be a sustainable resource for sighting endemic species in the area. Among the species observed while snorkeling are manta rays, hammerhead sharks, and seahorses. Fishing in the Galapagos is catch-and-release, where boats operated by captains and sailors authorized by the government of Ecuador are used.

## 4. Methodology

The present study is part of the Project that was approved ethically by the Espírtu Santo University of Ecuador. Informed consent was requested in writing at the beginning of the questionnaire. This study designed a questionnaire that would be used to collect the information that would be analyzed to obtain reliable results to achieve the proposed objectives. First, the existing literature that helped develop the questionnaire was analyzed. This instrument was designed in three parts. The first consisted of obtaining sociodemographic information and trip characteristics of the sample surveyed. This section comprised eight closed-type questions and was taken from the study by Lee et al. [27]. The second part of the instrument was developed to collect information about the recreational experiences of the sample and consisted of 17 items adapted from the study by Lee et al. [27]. The questions were asked on a 5-point Likert scale (1 is the least important, and 5 is very important). The Cronbach’s Alpha index of the recreational experiences scale was 0.921, which indicated high reliability between the scale items.

The third part of this questionnaire was about satisfaction and loyalty and was developed based on the study by Kim and Park [76]. The satisfaction questions were three on a 5-point Likert scale where (1 is very little satisfied and 5 is very satisfied). The loyalty questions had to do with the variables return, recommendation, and saying positive things about the destination and were prepared on a five-point Likert scale where (1 disagree and 5 strongly agree). After preparing the questionnaire, a group of experts reviewed and improved it. The questionnaire was prepared in English and Spanish. A pilot test of 30 surveys was carried out for one day with visitors recreating at ‘Mann Beach’ in San Cristóbal to correct errors in writing and compression.

The sample was collected from January to February 2019 at ‘Mann Beach,’ part of the marine protected area of San Cristóbal Island of the Galapagos Archipelago in Ecuador. Respondents should be over 18 years old and be national or foreign tourists. The Convenience Method was used because the tourist was chosen for his availability and accessibility. To reduce the bias of the method due to convenience, the diversity of the population was taken into account, so the samples were recognized on different days of the week, weekends, daytime hours and taking the sample in different geographical points of the destination which allowed us to reduce bias and increase the representativeness of the sample. This study had limitations in the generalization of the results, because the sample was taken in a specific destination.

The sample size was 407 valid questionnaires.

The sample size formula for infinite populations n = (z^2^.σ^2^)/e^2^ The confidence level was 95%, which corresponds to a Z value of 1.96. An error of 4.9% (less than 5%) was used and the population variance σ^2^ was set at 25%, as a conservative estimate where there is no prior information on the dispersion of the data. The collected data was analyzed using the statistical program SPSS, version 22.

Factor Analysis was applied to reduce the data into a smaller number of unobserved variables called factors. Varimax rotation was used to order the factor loadings. Kaiser’s criterion was used to find the number of factors with eigenvalues greater than one. The KMO index and Bartlett’s test of sphericity were used to determine whether the analysis was appropriate. Limitations of exploratory factor analysis include that the interpretation of factors is often subjective and context-dependent. This lack of objectivity can introduce bias into the conclusion of the analysis. Sometimes, the results are specific to the data set analyzed and may not be generalized to other contexts or populations.

In addition, a K-means cluster analysis has been used to group cases based on the distances between them in a set of variables. One of the limitations of the K-means technique is knowing the appropriate number of segments. The dendrogram was used to determine the optimal number of segments to use in the k-means analysis. The Kruskal-Wallis H test was used to verify the existence of the differences in the means between the three groups, and the Mann-Whitney U test was used to identify where these differences were between the means. Likewise, the Chi-square test was used to analyze the relationships between the segments and the satisfaction and loyalty variables.

## 5. Results

### 5.1. Sample profile

The tourist sample comprised 55.8% women and 44% men. Regarding marital status, the majority, 59.2%, were single. Regarding age, the majority, 42.1%, were between 21 and 30 years old. 62.9% had a university level. 30.7% were private employees, and 25.8% were students. 26.3% of those surveyed traveled alone, while 26% traveled with their partner. 27.3% had income between $1,001 to $1,500 monthly and 22.6% between $501 to $1,000 monthly. 33.7% spent between $50.01 - $100 per day, and 29.5% spent between $101.01 - $150 per day.

### 5.2. Dimensions of the recreational experience in coastal and marine destinations

A factor analysis has been used to interpret the items better and reduce them to fewer factors. The number of factors was found through eigenvalues greater than 1. Cronbach’s Alpha in each factor had values between 0.815 and 0.891, indicating a high internal correlation in each factor, giving the model a certain degree of robustness. The KMO index gave a result of 0.853 (value close to 1), and Bartlett’s test of sphericity was significant, so it was appropriate to perform the factor analysis model. See Table 1.

**Table 1:** Dimensions of the recreational experience in coastal and marine destinations.

Variable	Ecological Observation	Experiential Learning	Experiential Reflection	Sensory Experience
Get excited to see live animals	0.888			
I have a good view of animals	0.878			
Lots of activity to see or do	0.792			
Interest in the tourist experience developed in Galapagos	0.577			
Real learning experience		0.831		
It made me learn a lot		0.831		
It made me more knowledgeable		0.745		
Stimulate curiosity to learn new things		0.732		
Mentally challenging destination			0.804	
It allows to reflect and generate new ideas			0.732	
It allows to talk and share new information with colleagues			0.591	
Enjoyment of the tourist experience developed in Galapagos			0.561	
Sense of surprise or amazement			0.560	
The visit has generated a sense of harmony in me				0.772
It has been or is it nice just to be here				0.748
It allows to generate or inspire creative thoughts				0.654
Being here is appealing				0.621
Cronbach’s alpha	0.882	0.891	0.828	0.815
Eigenvalue	7.866	1.724	1.412	1.213
Variance explained (%)	46.268	10.140	8.305	7.134
Cumulative variance explained (%)	46.268	56.408	64.713	71.848

According to the results in Table 1, the first factor was related to the excitement of observing animals and having an excellent view of them. Therefore, this dimension has been labeled as “Ecological Observation.” This first dimension accounted for 46.27% of the explained variance. In contrast, the second dimension was “Experiential Learning” because it was associated with gaining more knowledge and learning a lot about new things. This factor contained 10.14% of the explained variance. The third factor was related to reflection, surprise, or amazement and was therefore called “Experiential Reflection.” This dimension included 8.31% of the explained variance.

Meanwhile, the fourth factor was named “Sensory Experience” because it was related to the pleasant sense of harmony and attractiveness generated by the visit. This dimension accounted for 7.13% of the explained variance. These results address our first research question: RQ1: What are the dimensions of the recreational experience in coastal and marine destinations focused on island marine protected areas?

### 5.3. Segmentation of the recreational experience in coastal and marine destinations

A segmentation of the recreational experience was carried out, for which the non-hierarchical K-means analysis was used. See Table 2.

**Table 2:** Segmentation based on recreational experiences in coastal and marine destinations.

Variable	Aestheticians	Recreational Multi-Experiencers	Hedonists	Kruskal Wallis	Sig.	U of Mann-Whitney
Lots of activity to see or do	4.05	4.50	1.98	146.45	0.000	All
I have a good view of animals	4.58	4.67	2.32	155.64	0.000	All except 1–2
Get excited to see live animals	4.67	4.69	2.13	176.76	0.000	All except 1–2
Interest in the tourist experience developed in Galapagos	3.75	4.43	2.32	140.41	0.000	All
Enjoyment of the tourist experience developed in Galapagos	3.55	4.44	2.51	144.61	0.000	All
Sense of surprise or amazement	3.67	4.54	2.72	123.54	0.000	All
Mentally challenging destination	2.61	4.30	2.81	150.47	0.000	All except 1–3
It allows to reflect and generate new ideas	3.19	4.47	2.91	145.13	0.000	All except 1–3
It allows to talk and share new information with colleagues	2.96	4.44	3.00	145.80	0.000	All except 1–3
It allows to generate or inspire creative thoughts	3.12	4.49	2.98	146.47	0.000	All except 1–3
The visit has generated a sense of harmony in me	3.60	4.56	3.23	94.13	0.000	All except 1–3
It has been or is it nice just to be here	4.10	4.64	3.17	92.06	0.000	All
Being here is appealing	3.85	4.68	3.17	105.41	0.000	All
It made me more knowledgeable	3.52	4.64	3.15	146.50	0.000	All except 1–3
It made me learn a lot	3.60	4.67	3.19	143.07	0.000	All except 1–3
Real learning experience	3.49	4.63	3.17	149.65	0.000	All except 1–3
Stimulate curiosity to learn new things	3.47	4.62	3.13	137.88	0.000	All except 1–3

According to the results of Table 2, the first segment was Aestheticians with high levels only because of the sensory experience (how pleasant and attractive the marine protected area is). The second group was called Multi-Experience Recreationists, for having high scores in all the recreational experiences that can be done in the marine protected area (sensory experience, learning, reflection, and ecological observation). In contrast, the third group was called Hedonists for having low scores in recreational experiences in the marine protected area. Results that answer our second research question: RQ2: What are the segments of the recreational experience in coastal and marine destinations focused on island marine protected areas?

### 5.4. Segmentation by recreation experiences in coastal and marine destinations and the variables of satisfaction and loyalty

Pearson’s Chi-square test was used to find significant differences between the segmentation variable and other variables such as satisfaction, intentions to return, intentions to recommend, and to say positive things about the marine protected area. See Table 3.

**Table 3:** Segmentation by recreation experiences and the variables of satisfaction and loyalty.

Variable	Aestheticians	Recreational Multi-Experiencers	Hedonists	Pearson’s Chi-square	Sig.
Satisfaction compared to previous expectations	4.37	4.50	3.11	137.817	0.000
I intend to come back to this destination	3.69	4.23	2.85	73.652	0.000
I intend to recommend this destination	4.47	4.58	3.09	114.277	0.000
I will say positive things when I talk about Galápagos	4.49	4.65	3.64	78.212	0.000

According to the results in Table 3, all three segments showed significant differences (p<0.05) with satisfaction and loyalty variables (intentions to return, intentions to recommend, and intentions to speak positively about the destination). Based on the results, the “Recreational Multi-Experiencers” segment had the highest scores in satisfaction and intentions to return, recommend, and speak positively about the marine protected area among the three groups. For this reason, this segment is crucial to satisfy, and it should receive greater attention. Service quality should be improved in all recreational activities to increase the satisfaction of this segment and encourage them to return to the destination, benefiting the community. On the other hand, the “Aestheticians” segment also had high scores in satisfaction and loyalty variables. Therefore, service quality related to the pleasant and attractive aspects of the marine protected area should be improved to maintain or enhance the satisfaction level of this segment.

Furthermore, tourists need to recommend the marine protected area. Service quality in all recreational activities should be improved to enhance tourists’ recommendations, especially from the “Recreational Multi-Experiencers” segment, as they enjoy all recreational activities in the marine protected area. These results address our third research question: RQ3: What is the relationship between recreational experience segments and satisfaction and loyalty in coastal and marine destinations focused on island marine protected areas?

## 6. Discussion

As its first objective, the present study had to identify the dimensions of recreational experiences in coastal and marine destinations focused on island marine protected areas. In response to RQ1, the results show that recreational experiences in coastal and marine destinations focused on island marine protected areas consist of four dimensions: Ecological Observation, Experiential Learning, Experiential Reflection, and Sensory Experience. In this regard, by reviewing previous findings, we can establish that our Ecological Observation factor was similarly found as “Ecological Observation” by Carvache-Franco et al. [10]. In contrast, Lee et al. [27] identified it as part of a dimension called “Reflective Experience and Ecological Observation.” Our Experiential Learning factor was identified similarly by Carvache-Franco et al. [10] and Lee et al. [27]. Our Experiential Reflection factor was found to be similar to Experiential Reflection by Carvache-Franco et al. [10], while Lee et al. [27] identified it as “Reflective Experience and Ecological Observation.” Our Sensory Experience factor was found similarly by Carvache-Franco et al. [10] as “Sensory and Experiential,” and Lee et al. [27] referred to it as “Experiential Sensory.” The contribution to the literature lies in being the first to identify these recreational experience factors applied to an island marine protected area. In this sense, the study provides four dimensions of recreational experiences, which have been similarly identified by other authors like Carvache-Franco et al. [10] and Lee et al. [27].

As a second objective, this research aimed to determine the demand segments for recreational experiences in coastal and marine destinations focused on island marine protected areas. In response to RQ2, the results reveal three distinct segments: Aestheticians, Multi-experience Recreationists, and Hedonists. In previous studies from academic literature, authors like Lee et al. [27] found multi-experience recreationists, aestheticians, hedonists, and knowledge seekers, which means they identified an additional group searching for knowledge. Similarly, Carvache-Franco et al. [10] found multiple-experience recreationists, hedonists, knowledge seekers, and aestheticians. A contribution to the academic literature is to identify these segments based on recreational activities in coastal and marine destinations focused on island marine protected areas.

As a third objective, this study aimed to establish the relationship between the demand segments for recreational experiences and satisfaction and loyalty in coastal and marine destinations focused on island marine protected areas. In response to RQ3, the results indicate that the segment of Multi-Experience Recreationists was the most satisfied and showed higher levels of loyalty in island marine protected areas. Similarly, Carvache-Franco et al. [10] found that the Multiple Experience Recreationist group was the most satisfied and loyal. Therefore, a contribution to scientific literature is the identification that, in a coastal and marine destination that is part of an island marine protected area, tourists belonging to the Multi-Experience Recreationists group are the ones who tend to be the most satisfied and loyal regarding recreational experiences.

As practical implications, it is recommended that to improve the Ecological Observation factor, flora and fauna sighting tours mixed with environmental education could be implemented. In the same way, to improve the experiential learning dimension, workshops could be given on the elaboration of handicrafts, local gastronomy, environmental care and community habits. Also, to increase the Experiential Reflection factor, visits to communities could be implemented, practicing local work, creating similar interest groups, experiential tourism, and implementing workshops to share experiences. Likewise, to improve the Sensory Experience factor, it would be necessary to implement workshops on community knowledge, courses for them to meet friends, reflective workshops on the life of species and environmental care, and courses on coastal sports activities.

Regarding segmentation, it is recommended that the sensory dimension and the visit attractiveness must be improved by creating recreational activities to attract the Aestheticians group. It is advisable to attract Multi-Experience Recreationist tourists because they have the highest satisfaction and loyalty to the destination. Therefore, it is recommended to offer tourism packages with various activities such as wildlife viewing, community visits, experiential tourism, environmental education, water sports, and coastal recreational activities. Additionally, to attract Hedonist tourists who have low scores in recreational experiences, it is recommended to involve them in all activities offered to multi-experience tourists and in social activities to increase their experience levels during their visit. In this sense, the results of this study can serve as management guidelines for managers of coastal and marine island destinations that are protected areas and for recreational tourism service providers to adapt their products according to the identified demand.

## 7. Conclusion

Recreational activities in the coastal and marine destinations that are part of the island marine protected areas include flora and fauna sighting, recreational navigation, experiential tourism, visits to museums, educational workshops, communities, recreational beach activities, and coastal and marine sports. Establishing the factors and segments encompassing tourists’ experiences when carrying out these activities is crucial to meet the demand and create sustainable development plans in these destinations.

Recreational experiences and activities are an essential component of coastal and marine tourism and contribute to the growth and sustainability of this sector. However, if not appropriately managed, recreational activities can negatively impact the natural environment and local communities. Therefore, it is essential to develop effective management strategies that balance the needs of recreational activities with conservation objectives and community interests. Recreational activities are a significant component of marine protected areas and can provide economic and social benefits. Understanding these factors can help destination managers and tourism stakeholders design and develop tourism products that meet the needs and preferences of tourists. Additionally, addressing environmental and social issues related to tourism development can help ensure the industry’s sustainability and protect coastal and marine areas’ natural and cultural resources. However, sustainable management practices are essential to ensure the long-term viability of the tourism industry in these destinations.

In this regard, the results show that the recreational experience in coastal and marine destinations focused on insular marine protected areas comprises four dimensions: Ecological observation, Experiential learning, Experiential reflection, and Sensory experience. Also, it has been identified that the demand comprises three differentiated segments, the first of which is Aestheticians, characterized by only enjoying the sensory experience, how pleasant and attractive the island marine protected area can be. The second segment is formed by the so-called multi-experience Recreationists, represented by enjoying all the recreational experiences in marine protected areas. At the same time, the third segment has been called Hedonists, as it is a group with less enjoyment of the recreational experience in marine protected areas. Of these groups, the Multi-Experience Recreationalists segment was the most satisfied and highly loyal in the island marine protected areas.

Regarding the theoretical implications, it has been identified that in coastal and marine destinations focused on insular marine protected areas, four factors characterize the demand concerning recreational experiences: ecological observation, experiential learning, experiential reflection, and sensory experience. Three segments of demand have also been found based on their recreational experiences in these destinations that are declared marine protected areas, which are Aestheticians, Multi-experience Recreationists, and Hedonists. Likewise, it has been determined that multi-experience Recreational tourists have the most excellent satisfaction and loyalty in the destinations in variables such as return, recommendation, and saying positive things about the destination. All of these are contributions to the scientific literature in the area of coastal and marine tourism.

Regarding practical implications, the present results will be important information for marine-protected area administrators to create management plans for the destination’s sustainability. Likewise, the community can align with the demand for recreational experiences and create products that match their stay preferences in the destination. By considering the social, environmental, psychological, and physiological dimensions of the recreational experience, researchers and professionals can develop more effective and satisfying recreational experiences for individuals and groups. Measuring these dimensions is essential for the practical assessment and design of recreational experiences that meet the needs and preferences of participants. Understanding these dimensions or factors is fundamental for the development and management of recreational and tourism activities, as well as for increasing visitor satisfaction and promoting repeat visits to recreational destinations. Researchers can identify different consumer segments by leveraging various segmentation criteria and methodologies, allowing companies to develop personalized marketing strategies and product offerings that enhance customer satisfaction and loyalty.

Although this work is innovative for being a study that had not been conducted before, it also has limitations, such as the timing of the sample collection because demand can vary, and therefore, the results. Another limitation is the method applied, which is convenience sampling and, therefore, non-probabilistic. Finally, a study related to the relationship between sociodemographic aspects and recreation experience factors is recommended as a future research direction.

## Supporting information

S1 Data (XLSX)

## References

[1] Mestanza-Ramón C., Capa M. S., Saavedra H. F., & Paredes J. R. Integrated coastal zone management in continental Ecuador and Galapagos Islands: Challenges and opportunities in a changing tourism and economic context. Sustainability, 2019; 11(22), 6386. doi: 10.3390/su11226386

[2] Sala E., & Giakoumi S. No-take marine reserves are the most effective protected areas in the ocean. ICES Journal of Marine Science, 2018;75(3), 1166–1168. doi: 10.1093/icesjms/fsx059

[3] Stevenson S. L., Woolley S. N., Barnett J., & Dunstan P. Testing the presence of marine protected areas against their ability to reduce pressures on biodiversity. Conservation Biology, 2020;34(3), 622–631. doi: 10.1111/cobi.13429 31667866

[4] MedPAN. The 2016 status of Marine Protected Areas in the Mediterranean: Main findings. Brochure MedPAN & UN Environment/MAP ‐ SPA/RAC. Available at https://medpan.org/sites/default/files/media/downloads/medpan-forum_mpa_2016_-_brochure_a4_en_web.pdf

[5] Frauman E. & Norman WC. Managing Visitors via" Mindful" Information Services: One Approach in Addressing Sustainability. Journal of Park & Recreation Administration, 2003; 21(4). https://js.sagamorepub.com/index.php/jpra/article/view/1493

[6] Jaafar M. & Maideen SA. Ecotourism-related products and activities, and the economic sustainability of small and medium island chalets. Tourism Management. 2012. doi: 10.1016/j.tourman.2011.07.011

[7] Carvache-Franco W., Carvache-Franco M. & Hernández-Lara AB From motivation to segmentation in coastal and marine destinations: a study from the Galapagos Islands, Ecuador, Current Issues in Tourism, 2021;24:16, 2325–2341, doi: 10.1080/13683500.2020.1811651

[8] Lee TH, Jan FH & Huang GW. The influence of recreation experiences on environmentally responsible behavior: The case of Liuqiu Island, Taiwan. Journal of Sustainable tourism, 2015; 23(6), 947–967. doi: 10.1080/09669582.2015.1024257

[9] Prayag G. & Ryan C. Antecedents of tourists’ loyalty to Mauritius: The role and influence of destination image, place attachment, personal involvement, and satisfaction. Journal of travel research, 2012; 51(3), 342–356. doi: 10.1177/0047287511410321

[10] Carvache-Franco M.; Carvache-Franco W., & Manner- Baldeon F., Market segmentation based on ecotourism motivations in marine protected areas and National Parks in the Galapagos Islands, Ecuador. Journal of Coastal Research, 2021; 37(3), 620–633. Coconut Creek (Florida), ISSN 0749-0208. 10.2112/JCOASTRES-D-20-00076.1

[11] Schubert SF, Brida JG & Risso WA. The impacts of international tourism demand on economic growth of small economies dependent on tourism. Tourism Management, 2011; 32(2), 377–385. doi: 10.1016/j.tourman.2010.03.007

[12] Kuo NW & Chen PH. Quantifying energy use, carbon dioxide emission, and other environmental loads from island tourism based on a life cycle assessment approach. Journal of cleaner production, 2009; 17, 1324–1330. doi: 10.1016/j.jclepro.2009.04.012

[13] Moyle B., Weiler B. & Croy G. Visitors’ perceptions of tourism impacts: Bruny and Magnetic Islands, Australia. Journal of Travel Research, 2013;52(3), 392–406. doi: 10.1177/0047287512467702

[14] UNWTO (2017). The year of sustainable tourism for development. https://webunwto.s3.eu-west-1.amazonaws.com/s3fs-public/2021-03/iy_roadmap_en_web.pdf?VersionId=XPYzDve4xH5Zpw_SFN71RrPandtsH5MS

[15] Waayers D., Lee D., Newsome D. Exploring the nature of stakeholder collaboration: A case study of marine turtle tourism in the Ningaloo region, Western Australia. Current Issues in Tourism, 2012;15(7), 673–692., doi: 10.1080/13683500.2011.631697

[16] Hall CM, Gössling S. & Scott D. The Routledge handbook of tourism and sustainability. Routledge, 2015. https://www.researchgate.net/publication/283638225_Handbook_of_Tourism_and_Sustainability

[17] Ballantyne R., Packer J. & Sutherland LA Visitors’ memories of wildlife tourism: Implications for the design of powerful interpretive experiences. Tourism Management, 2011; 32(4), 770–779. doi: 10.1016/j.tourman.2010.06.012

[18] Driver BL, Brown PJ & Peterson GL Benefits of leisure. In Preliminary drafts of the chapters in this volume were presented at a workshop of the authors in Snowbird, Utah, May 1989. Venture Publishing. Available at https://psycnet.apa.org/record/1992-97201-000

[19] Larson R., Csikszentmihalyi M. The Experience Sampling Method. Flow and the Foundations of Positive Psychology, 2014. Springer, Dordrecht. doi: 10.1007/978-94-017-9088-8_2

[20] Kline C., Hao H., Alderman D., Kleckley J. W., & Gray S. A spatial analysis of tourism, entrepreneurship and the entrepreneurial ecosystem in North Carolina, USA. Tourism Planning & Development, 2014; 11(3), 305–316. Available at https://www.tandfonline.com/doi/abs/10.1080/21568316.2014.890127

[21] Newsome D. y Dowling RK. The scope and nature of geotourism. Geotourism, 2005;3, 25, Routledge, London. Available at https://www.taylorfrancis.com/chapters/edit/10.4324/9780080455334-2/scope-nature-geotourism-david-newsome-ross-dowling

[22] Maldonado-Ore EM & Custodio M. Visitor environmental impact on protected natural areas: An evaluation of the Huaytapallana Regional Conservation Area in Peru. Journal of Outdoor Recreation and Tourism, 2020;31, 100298. doi: 10.1016/j.jort.2020.100298

[23] Gurney GG, Pressey RL, Ban NC, Álvarez‐Romero JG, Jupiter S. & Adams VM Efficient and equitable design of marine protected areas in Fiji through inclusion of stakeholder‐specific objectives in conservation planning. Conservation Biology, 2015;29(5), 1378–1389. doi: 10.1111/cobi.12514 25916976

[24] Munanura IE, Needham MD, Lindberg K., Kooistra C. & Ghahramani L. Support for tourism: The roles of attitudes, subjective wellbeing, and emotional solidarity. Journal of Sustainable Tourism, 2023; 31(2), 581–596. doi: 10.1080/09669582.2021.1901104

[25] Munar AM & Jacobsen JK Motivations for sharing tourism experiences through social media. Tourism management, 2014; 43, 46–54. doi: 10.1016/j.tourman.2014.01.012

[26] Buckley R., Pickering C. & Weaver D. Nature-based tourism, environment and land management, 2003. CABI Editorial. doi: 10.1079/9780851997322.0000

[27] Lee TH, Jan FH, Tseng CH & Lin YF. Segmentation by recreation experience in island-based tourism: A case study of Taiwan’s Liuqiu Island. Journal of Sustainable tourism, 2018; 26(3), 362–378. doi: 10.1080/09669582.2017.1354865

[28] Öhman S. Previous experiences and risk perception: The role of transference. Journal of Education, Society and Behavioural Science, 2017;23(1), 1–10. doi: 10.9734/JESBS/2017/35101

[29] Huh J., Uysal M. & McCleary K. Cultural/heritage destinations: Tourist satisfaction and market segmentation. Journal of Hospitality & Leisure Marketing, 2006;14(3), 81–99. doi: 10.1300/J150v14n03_07

[30] Richards, G. (Ed.). Cultural tourism: Global and local perspectives. Psychology Press, 2007. Available at https://books.google.es/books?hl=es&lr=&id=-LXgdr3hbkgC&oi=fnd&pg=PR13&dq=Richards,+G.+(Ed.).+(2007).+Cultural+tourism:+Global+and+local+perspectives.+Psychology+Press.&ots=QHor4sv-OF&sig=5FlBthA9RuMozQwTW7RQVfYD2v0#v=onepage&q=Richards%2C%20G.%20(Ed.).%20(2007).%20Cultural%20tourism%3A%20Global%20and%20local%20perspectives.%20Psychology%20Press.&f=false

[31] Hall CM & Sharples L. The consumption of experiences or the experience of consumption? An introduction to the tourism of taste. Food tourism around the world, 2004; (pages. 1–24). Routledge. Available at https://www.taylorfrancis.com/chapters/edit/10.4324/9780080477862-1/consumption-experiences-experience-consumption-introduction-tourism-taste-michael-hall-liz-sharples

[32] Sims R. Food, place and authenticity: local food and the sustainable tourism experience. Journal of sustainable tourism, 2009;17(3), 321–336. doi: 10.1080/09669580802359293

[33] Rheeders T. A review of the determinants of tourism destination competitiveness. Journal of Contemporary Management, 2022;19(2), 238–268. doi: 10.35683/jcman1008.166

[34] Postma A. & Schmuecker D. Understanding and overcoming negative impacts of tourism in city destinations: conceptual model and strategic framework. Journal of Tourism Futures. 2017;3(2), 144–156. https://www.emerald.com/insight/content/doi/10.1108/JTF-04-2017-0022/full/html

[35] Buhalis D. & Law R. Progress in information technology and tourism management: 20 years on and 10 years after the Internet—The state of eTourism research. Tourism Management, 2008;29(4), 609–623. doi: 10.1016/j.tourman.2008.01.005

[36] Lee CK, Olya H., Ahmad MS, Kim KH & Oh MJ. Sustainable intelligence, destination social responsibility, and pro-environmental behaviour of visitors: Evidence from an eco-tourism site. Journal of Hospitality and Tourism Management, 2021;47, 365–376. doi: 10.1016/j.jhtm.2021.04.010

[37] Dwyer L., Forsyth P. & Spurr R. Assessing the economic impacts of events: A computable general equilibrium approach. Journal of travel research, 2006;45(1), 59–66. doi: 10.1177/0047287506288907

[38] Manning RE. Studies in outdoor recreation: Search and research for satisfaction. Oregon State University Press, 2011. https://muse.jhu.edu/pub/205/monograph/book/111096

[39] Leisch F., Dolnicar S. & Grün B. Market segmentation analysis: Understanding it, doing it, and making it useful, 2018. https://library.oapen.org/handle/20.500.12657/51281

[40] Kotler P. & Keller KL Marketing management. 15th edition. Pearson Education Ltd, 2012. https://www.edugonist.com/wp-content/uploads/2021/09/Marketing-Management-by-Philip-Kotler-15th-Edition.pdf

[41] Carvache-Franco M., Segarra -Oña M., & Carrascosa-López C. Segmentation by motivation in ecotourism: Application to protected areas in Guayas, Ecuador. Sustainability, 2019;11(1), 240. doi: 10.3390/su11010240

[42] Almeida A. M. M., Correia A., & Pimpão A. Segmentation by benefits sought: the case of rural tourism in Madeira. Current Issues in Tourism, 2014;17(9), 813–831. https://www.tandfonline.com/doi/abs/10.1080/13683500.2013.768605

[43] Kozak M. & Rimmington M. Tourist satisfaction with Mallorca, Spain, as an off-season holiday destination. Journal of travel research, 2000;38 (3), 260–269. doi: 10.1177/004728750003800308

[44] Alegre J., & Garau J. Tourist satisfaction and dissatisfaction. Annals of Tourism Research, 2010;37(1), 52–73. doi: 10.1016/j.annals.2009.07.001

[45] Loncaric D., Dlacic J. & Bagaric L. Exploring the Relationship Between Satisfaction with Tourism Services, Revisit Intention and Life Satisfaction. Economic and Social Development: Book of Proceedings, 2019;122–132. https://esd-conference.com/upload/book_of_proceedings/Book_of_Proceedings_esdBelgrade2019_Online.pdf#page=129https://www.wir.ue.wroc.pl/info/book/WUT229c81011f16460fb67a29e0275f9e3a/

[46] Oliver RL. Satisfaction: A behavioral perspective on the consumer: A behavioral perspective on the consumer. Routledge, 2014. doi: 10.4324/9781315700892

[47] Oliver RL. A cognitive model of the antecedents and consequences of satisfaction decisions. Journal of marketing research, 1980;17 (4), 460–469. doi: 10.1177/002224378001700405

[48] Baker DA & Crompton JL. Quality, satisfaction and behavioral intentions. Annals of Tourism Research, 2000;27 (3), 785–804. doi: 10.1016/S0160-7383(99)00108-5

[49] Chen CF & Chen FS. Experience quality, perceived value, satisfaction and behavioral intentions for heritage tourists. Tourism management, 2010;31 (1), 29–35. doi: 10.1016/j.tourman.2009.02.008

[50] Wei Z., Zhang M. & Ming Y. Understanding the effect of tourists’ attribute-level experiences on satisfaction–a cross-cultural study leveraging deep learning. Current Issues in Tourism, 2022;26(1), 105–121. doi: 10.1080/13683500.2022.2030682

[51] Cutler SQ & Carmichael BA. The dimensions of the tourist experience. The tourism and leisure experience: Consumer and managerial perspectives, 2010;44, 3–26. doi: 10.21832/9781845411503-004

[52] Hosany S. & Gilbert D. Measuring tourists’ emotional experiences toward hedonic holiday destinations. Journal of travel research, 2010;49 (4), 513–526. doi: 10.1177/0047287509349267

[53] Hosany S., Prayag G., Deesilatham S., Cauševic S. & Odeh K. Measuring tourists’ emotional experiences: Further validation of the destination emotion scale. Journal of Travel Research, 2015;54 (4), 482–495. doi: 10.1177/0047287514522878

[54] Dolnicar S. & Otter T. Which hotel attributes matter? A review of previous and a framework for future research. Proceedings of the 9th Annual Conference of the Asia Pacific, 2003;176–188. https://ro.uow.edu.au/commpapers/268/

[55] Akbaba A. Measuring service quality in the hotel industry: A study in a business hotel in Turkey. International journal of hospitality management, 2006;25 (2), 170–192. doi: 10.1016/j.ijhm.2005.08.006

[56] Wu X., Gursoy D. & Zhang M. Effects of social interaction flow on experiential quality, service quality and satisfaction: moderating effects of self-service technologies to reduce employee interruptions. Journal of Hospitality Marketing & Management, 2021;30(5), 571–591. doi: 10.1080/19368623.2021.1867284

[57] Reisinger Y. & Turner L. Cross-cultural behaviour in tourism. Routledge, 2012. https://www.taylorfrancis.com/books/mono/10.4324/9780080490861/cross-cultural-behaviour-tourism-yvette-reisinger-phd-lindsay-turner

[58] Yoon Y. & Uysal M. An examination of the effects of motivation and satisfaction on destination loyalty: a structural model. Tourism management, 2005;26(1), 45–56. doi: 10.1016/j.tourman.2003.08.016

[59] Prayag G., Hosany S. & Odeh K. The role of tourists’ emotional experiences and satisfaction in understanding behavioral intentions. Journal of Destination Marketing & Management, 2013;2 (2), 118–127. doi: 10.1016/j.jdmm.2013.05.001

[60] Oh S., Ji H., Kim J., Park E. & Del Pobil AP. Deep learning model based on expectation-confirmation theory to predict customer satisfaction in hospitality service. Information Technology & Tourism, 2022;24 (1), 109–126. https://link.springer.com/article/10.1007/s40558-022-00222-z

[61] Parasuraman A., Zeithaml VA & Berry LL. A conceptual model of service quality and its implications for future research. Journal of marketing, 1985;49 (4), 41–50. doi: 10.1177/002224298504900403

[62] Sever I. Importance-performance analysis: A valid management tool?. Tourism management, 2015;48, 43–53. doi: 10.1016/j.tourman.2014.10.022

[63] Phadermrod B., Crowder RM & Wills GB. Importance-performance analysis based SWOT analysis. International journal of information management, 2019;44, 194–203. doi: 10.1016/j.ijinfomgt.2016.03.009

[64] Preziosi M., Acampora A., Lucchetti MC & Merli R. Delighting hotel guests with sustainability: Revamping importance-performance analysis in the light of the three-factor theory of customer satisfaction. Sustainability, 2022;14 (6), 3575. doi: 10.3390/su14063575

[65] Rather RA, Hollebeek LD & Islam JU. Tourism-based customer engagement: The construct, antecedents, and consequences. The Service Industries Journal, 2019;39 (7–8), 519–540. doi: 10.1080/02642069.2019.1570154

[66] Prayag G., & Ryan C. Antecedents of tourists’ loyalty to Mauritius: The role and influence of destination image, place attachment, personal involvement, and satisfaction. Journal of travel research, 2012;51(3), 342–356. doi: 10.1177/0047287511410321

[67] Oppermann M. Tourism destination loyalty. Journal of travel research, 2000;39 (1), 78–84. doi: 10.1177/004728750003900110

[68] Meng F., Tepanon Y. & Uysal M. Measuring tourist satisfaction by attribute and motivation: The case of a nature-based resort. Journal of vacation marketing, 2008;14 (1), 41–56. doi: 10.1177/1356766707084218

[69] Chen CF & Tsai D. How destination image and evaluative factors affect behavioral intentions?. Tourism management, 2007;28 (4), 1115–1122. doi: 10.1016/j.tourman.2006.07.007

[70] Gallarza MG, Saura IG & García HC. Destination image: Towards a conceptual framework. Annals of tourism research, 2002;29 (1), 56–78. doi: 10.1016/S0160-7383(01)00031-7

[71] Litvin SW, Goldsmith RE & Pan B. Electronic word-of-mouth in hospitality and tourism management. Tourism management, 2008;29 (3), 458–468. doi: 10.1016/j.tourman.2007.05.011

[72] Gursoy D. & McCleary KW. An integrative model of tourists’ information search behavior. Annals of tourism research, 2004;31 (2), 353–373. doi: 10.1016/j.annals.2003.12.004

[73] Reichheld FF.The one number you need to grow. Harvard business review, 2003;81(12), 46–55. https://www.researchgate.net/publication/8927283_The_One_Number_you_Need_to_Grow 14712543

[74] Bowen JT & Shoemaker S. Loyalty: A strategic commitment. The Cornell Hotel and Restaurant Administration Quarterly, 2003;44 (5–6), 31–46. doi: 10.1177/001088040304400505

[75] Ministry of Tourism. Galapagos reached record numbers in traveler arrival, 2023. https://www.turismo.gob.ec/galapagos-alcanzo-cifras-record-en-la-llegada-de-viajeros/

[76] Kim W., & Park J. Examining structural relationships between work engagement, organizational procedural justice, knowledge sharing, and innovative work behavior for sustainable organizations. Sustainability, 2017;9(2), 205. doi: 10.3390/su9020205

